# Influence of Culture Substrates on Morphology and Function of Pulmonary Alveolar Cells In Vitro

**DOI:** 10.3390/biom11050675

**Published:** 2021-04-30

**Authors:** Chiara Emma Campiglio, Marina Figliuzzi, Sara Silvani, Matteo Tironi, Sara Conti, Federica Boschetti, Andrea Remuzzi

**Affiliations:** 1 Department of Management, Information and Production Engineering, University of Bergamo, 24044 Dalmine, Italy; chiaraemma.campiglio@unibg.it; 2 Department of Biomedical Engineering, Istituto di Ricerche Farmacologiche Mario Negri-IRCCS, 24126 Bergamo, Italy; marina.figliuzzi@marionegri.it (M.F.); sara.silvani@marionegri.it (S.S.); matteo.tironi@marionegri.it (M.T.); 3 Department of Molecular Medicine, Istituto di Ricerche Farmacologiche Mario Negri-IRCCS, 24126 Bergamo, Italy; sara.conti@marionegri.it; 4 Department of Chemistry, Materials and Chemical Engineering Giulio Natta, Politecnico di Milano, 20131 Milan, Italy; federica.boschetti@polimi.it

**Keywords:** cell function, cell morphology, mechanobiology, substrate properties, lung epithelial cells

## Abstract

Cell’s microenvironment has been shown to exert influence on cell behavior. In particular, matrix-cell interactions strongly impact cell morphology and function. The purpose of this study was to analyze the influence of different culture substrate materials on phenotype and functional properties of lung epithelial adenocarcinoma (A549) cells. A549 cells were seeded onto two different biocompatible, commercially available substrates: a polyester coverslip (Thermanox™ Coverslips), that was used as cell culture plate control, and a polydimethylsiloxane membrane (PDMS, Elastosil^®^ Film) investigated in this study as alternative material for A549 cells culture. The two substrates influenced cell morphology and the actin cytoskeleton organization. Further, the Yes-associated protein (YAP) and its transcriptional coactivator PDZ-binding motif (TAZ) were translocated to the nucleus in A549 cells cultured on polyester substrate, yet it remained mostly cytosolic in cells on PDMS substrate. By SEM analysis, we observed that cells grown on Elastosil^®^ Film maintained an alveolar Type II cell morphology. Immunofluorescence staining for surfactant-C revealing a high expression of surfactant-C in cells cultured on Elastosil^®^ Film, but not in cells cultured on Thermanox™ Coverslips. A549 cells grown onto Elastosil^®^ Film exhibited morphology and functionality that suggest retainment of alveolar epithelial Type II phenotype, while A549 cells grown onto conventional plastic substrates acquired an alveolar Type I phenotype.

## 1. Introduction

The lung alveoli are air sacs that get oxygen into bloodstream and take carbon dioxide out. The alveolar epithelium is a dynamic tissue normally undergoing a slow, but constant renewal [1]. It is predominantly composed of alveolar Type I (ATI) and Type II (ATII) epithelial cells. ATI cells are large squamous cells, with a flat shape, allowing gas exchange and unable to replicate [2]. ATII pneumocytes are cuboidal cells dispersed throughout the alveoli, with high metabolic activity, and represent 15% of total lung cells [3]. These cells divide, and their cell progeny is able to maintain morphologic characteristics of ATII cells or transdifferentiate in Type I pneumocytes contributing to the epithelium reparation upon injury [2]. Moreover, ATII pneumocytes basically secrete the surfactant proteins implied in the regulation of alveolar surface tension during gas exchange, in alveolar fluid balance and in host defense [4,5].

Isolated ATII pneumocytes cultured in vitro onto conventionally used plastic substrates lose their specific features and acquire ATI epithelial cell characteristics, stopping surfactant production [6]. Continuous cell lines are an alternative to primary ATII epithelial cell cultures. A representative cell system used to investigate ATII epithelial cells is the A549 lung epithelial adenocarcinoma cell line [7,8]. The advantages of A549 cells are that they grow indefinitely and are easy to maintain in culture [9]. These cells show a morphology and a distribution of surfactant containing lamellar bodies in line with ATII cells of the pulmonary epithelium, as well as metabolic and transport properties consistent with these cells [10]. A549 cells are usually cultured in conventional two-dimensional (2D) systems based on adherent cell monolayers in a static dish culture. Under such conditions, A549 cells flatten, lose differentiated Type II cell morphologic characteristic, and stop synthesis and secretion of biochemical markers of surfactant [2]. These metabolic changes already occur within the first 24 h of culture and are progressive over several days in culture [11,12]. Despite these phenotypic changes, conventional 2D culture systems are largely used for toxicological investigations [13,14], as an alternative to animal studies.

It is well known that the cellular phenotype and function is dependent on several factors including chemical, physical and mechanical stimuli [15]. Numerous studies highlight the transduction of physical forces into biochemical signals, implying responses even at a single cell level [16,17,18]. Cells recognize the rigidity of their surrounding environment (extracellular matrix, ECM) and respond to mechanical cues through a number of mechanisms, many of them mediated by transmembrane proteins (i.e., integrins) [19,20]. Pathological processes, associated with changes in biomechanical properties of ECM, influence cell behaviors and the progression of several diseases such as lung cancer, lung inflammation and pulmonary fibrosis [21,22,23,24]. Particularly, in the case of Type II alveolar cells, mechanical forces due to the elasticity of alveolar ECM have a crucial role in maintenance of biochemical, morphological and molecular expression of Type II cell phenotype [25]. Alveolar epithelial cells cultured on increasingly stiff substrates developed an improved contractility, leading to activation of transforming growth factor beta (TGF-β) [26].

Cytoskeleton organization influences also the transduction of important cell transcriptional regulators. Recently, a growing body of literature has focused its attention on the emerging role of Yes-associated protein (YAP) and its transcriptional coactivator PDZ-binding motif (TAZ) in mechanotransduction. YAP/TAZ are the main transcriptional effector of the Hippo signaling pathways involved in tumor growth, cellular proliferation and inhibition of apoptotic signals [27,28,29]. In epithelial cells, YAP and TAZ act as sensors of epithelial cell polarity being inhibited when cells differentiate an apical membrane domain, and being activated when cells come into contact with the extracellular matrix via their basal membrane domain [30]. Mechanical and cytoskeletal inputs regulate YAP/TAZ nuclear accumulation and activation, which has been associated with significant biological effect in epithelial cells. Increased YAP activity in airway basal stem cells leads to epithelial hyperplasia and impairs terminal differentiation, whereas YAP deletion determines either terminal differentiation or loss of basal cells ability to dedifferentiate into progenitor cells [31,32]. Elbediwy and coworkers showed that nucleus-localized YAP is required in skin keratinocytes to induce cell proliferation [33]. Similarly, in epithelial cells in lungs of patients with idiopathic pulmonary fibrosis, YAP nuclear translocation regulates cell migration, proliferation and polarity [34].

All the data reported above suggest that the properties of cell substrate may importantly affect cell structure and function. Thus, the aim of our work was to investigate the effects of two different substrates on the behavior of A549 cells in culture. Conventional cell culture substrate was compared to a PDMS membrane in a standard in vitro cell culture system to investigate cells response to a possible alternative substrate, that has interesting properties, such as high biocompatibility and availability, and it is sterilizable and ready-to-use. We analyzed alterations in cell morphology and cytoskeletal architecture of cells seeded on the two different substrates to evaluate the effects of substrate properties on A549 cell behavior. This investigation may be important in order to understand in which in vitro culture condition A549 cells are able to acquire and maintain physiological phenotypes.

## 2. Materials and Methods

### 2.1. Characterization of Substrates

Two different substrates were used for A549 cell culture: a rigid surface of cell culture treated Thermanox™ Coverslips (Thermo Fisher Scientific, Waltham, MA, USA, 200 μm thickness) and a polydimethylsiloxane (PDMS) artificial elastic membrane (Elastosil^®^ Film, Silex Ltd., Hampshire, UK, 100 μm thickness). To characterize the two substrates a tensile testing was carried out. Rectangular specimens of Thermanox™ Coverslips and Elastosil^®^ Film (*n* = 5 for each type of material) were mounted on a universal testing machine (MTS System Corporation, Eden Prairie, MN, USA)*,* gripped at their two ends and subjected to a controlled tension until failure, at a velocity of 0.1 mm/s. Stress was calculated by dividing the machine-measured force by the initial area (thickness times width). Strain was calculated by normalizing the machine actuator displacement by the specimen’s initial length measured between the machine grips. From the resulting stress–strain curves (σ–ε), the elastic modulus was derived.

To provide a more suitable substrate for cell adhesion, a fibronectin (FN) coating was performed on both substrates following an adsorption process [35]. In brief, sterile Thermanox and Elastosil samples were placed in 24-well plate and immersed in 1 mL of fibronectin solution (5 µg/mL) overnight at 4 °C. Afterwards, they were washed with phosphate buffer solution (PBS) and used as cell culture substrates. The presence of FN on both substrates was investigated by immunofluorescence staining. Samples (*n* = 2 for each condition) were immersed in PBS-BSA (Bovine Serum Albumin) saturation buffer 3% (*w*/*v*) for 30 min at room temperature. After saturation, samples were incubated for 45 min at room temperature with a solution of rabbit anti-fibronectin polyclonal antibody (diluted 1:500, Abcam, Cambridge, UK, ab2413). Then samples were rinsed two times with saturation buffer and incubated again for 45 min at room temperature with a solution of Donkey anti-rabbit IgG Cy3 conjugate secondary antibody (diluted 1:100, A32754, Jackson Immunoresearch, West Grove, PA, USA). Finally, samples were washed two times with saturation buffer and the fluorescence images were recorded by a laser confocal microscopy (Leica SP8).

### 2.2. Cells Culture and Experimental Design

Human lung adenocarcinoma cell lines A549 were obtained from the American Type Culture Collection (ATCC^®^ CCL-185™). A549 cells were routinely cultured in F12K medium (ATCC, Middlesex, UK) supplemented with 10% inactivated fetal bovine serum (Thermo Fisher Scientific). Cells were grown in T75 flasks at 37 °C with 5% CO_2_ and trypsinized for passage three times a week.

For cell culture experiments, A549 cells were seeded at concentration of 1 × 10^4^ cells/cm^2^ on Thermanox™ Coverslips and Elastosil^®^ Film, which had previously been coated with fibronectin (5 µg/mL). Cells were maintained in culture for 72 h. Every 24 h images were digitized using a phase-contrast microscope (Axiovert 40 C, Carl Zeiss Inc., Gottingen, Germany) equipped with a digital camera (PowerShot G5, Canon Inc., Tokyo, Japan) to monitor cell adherence and growth. After 3 days of culture, seeded substrates (3 samples for each condition, experiments were completed in duplicate) were incubated at 37 °C in 20% *v*/*v* resazurin solution (Sigma Aldrich, St. Louis, MO, USA) for metabolic activity quantification, using tissue culture polystyrene (TCPS) as control. After 2 h, 50 μL/sample were transferred to a 96-well plate and fluorescence (Ex/Em at 530 nm/590 nm) was measured in a microplate spectrophotometer (Infinite m200-pro, Tecan Group Ltd., Mannedorf, Switzerland).

### 2.3. Scanning Electron Microscopy Analysis

To evaluate three-dimensional shape and features of cells grown on Thermanox™ Coverslips and Elastosil^®^ Film, we analyzed our samples through scanning electron microscopy (SEM). Samples were fixed in 0.5% glutaraldehyde (Sigma Aldrich) in 0.1 M sodium cacodylate (Sigma Aldrich) for 1 h, then post fixed with osmium tetroxide (Società Italiana Chimici, Roma, Italy) and dehydrated through a series of passages in increasing ethanol baths. Then, cells were dried in pure hexamethyldisilane (HMDS, Fluka Chemie AG, Buchs, Switzerland). At the end, samples were mounted on stubs, coated with gold in a sputter coater (Agar Scientific, Stansted, UK) and then examined on a Cross-Beam 1540EsB electron microscope (Carl Zeiss GmbH, Oberkochen, Germany).

### 2.4. Immunofluorescence

In order to analyze the effect of substrate properties on A549 cells behavior, immunofluorescence analysis was performed. Briefly, cells were fixed in 2% paraformaldehyde (Società Italiana Chimici) in 4% sucrose (Sigma Aldrich), for 10 min at room temperature, permeabilized with 0.1% triton X-100 (Sigma Aldrich) in PBS and blocked with 3% BSA (Sigma Aldrich). Subsequently, cells were incubated overnight at 4 °C with different solutions of primary antibodies: (1) rabbit polyclonal anti-YAP-1 (diluted 1:1000, ab81183, Abcam, Cambridge, UK) to evaluate YAP-1 expression, (2) rabbit cleaved caspase-3 (diluted 1:200, product number 9661, Cell Signaling Technologies, Danvers, MA, USA) to study possible apoptosis, (3) rabbit polyclonal to Prosurfactant Protein C (diluted 1:200, ab90716, Abcam, Cambridge, UK) to investigate surfactant protein C expression and (4) rabbit polyclonal phospho-focal adhesion kinase (p-FAK, diluted 1:200, ab81183, Abcam, Cambridge, UK) to stain focal adhesions (FAs). Moreover, to study the conformation of actin filaments, samples were incubated with rhodamine-labeled phalloidin (diluted 1:40, Invitrogen, Paisley, UK) for 1 h at room temperature. The secondary antibody used to detect YAP-1 and Prosurfactant Protein C signals was Donkey anti-rabbit IgG Cy3 conjugate (diluted 1:100, A32754, Jackson Immunoresearch, West Grove, PA, USA), while caspase-3 and p-FAK were detected with FITC-conjugated anti-rabbit IgG (H + L) (diluted 1:25, F-2765, Jackson Immunoresearch), for 1 h at room temperature. Counterstaining with DAPI 1 μg/mL was performed for cell nuclear staining. Cells were finally examined with a laser confocal microscopy (Leica SP8).

Cell spread area was quantified on F-actin cytoskeleton images, using NIH Image J software. For each condition, 10 non-overlapping random images were analyzed (experiment was completed in duplicate).

A quantitative investigation of focal adhesions formed by A549 cells seeded on Thermanox™ Coverslips and Elastosil^®^ Film was performed analyzing the immunofluorescence 8-bit images acquired. All steps of the image processing were carried out using NIH Image J software. Briefly, the presence of focal adhesion and its dimension was measured considering the overlapping between actin filament and FA, that gives in the final image a signal in the yellow channel (5 non-overlapping random images for each condition were analyzed. Each experiment was completed in duplicate).

Quantification of nuclear YAP-1 was also evaluated on digitized images using NIH Image J software. The percentage of nuclear YAP-1 over the total YAP-1 positive signal was counted on at least 8 non-overlapping random images for each experiment (experiments were completed in duplicate). For each image, Regions of Interest (ROI) were selected on cell nuclei by using the DAPI signal. On the YAP signal image, the total YAP positive area was measured and then the selected ROI were applied in order to delimit nuclear versus cytosolic region and evaluate the corresponding percentages of YAP-1 signal.

### 2.5. Statistical Analysis

Data are expressed as mean ± standard deviation (SD). Datasets tested for normality show a normal distribution. Unpaired Student’s *t*-test and one-way ANOVA followed by Tukey’s multiple comparison test were adopted to estimate statistical significance between two and more group comparisons, respectively (Prism 8.0; GraphPad Software Inc., San Diego, CA, USA). Differences between groups were considered statistically significant when *p* < 0.05.

## 3. Results

### 3.1. Characterization of Substrates

**Figure 1:**
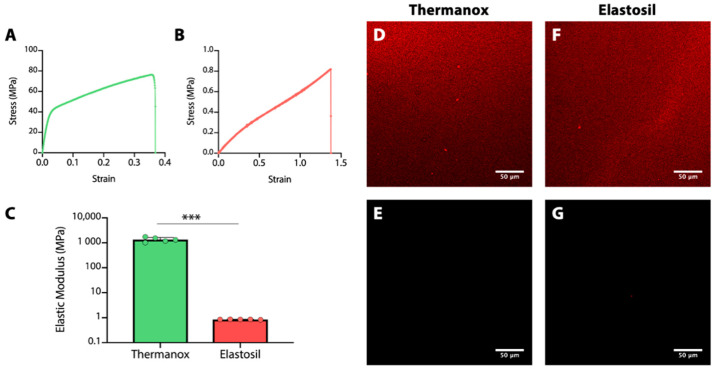
(**A**,**B**) Stress-strain curves obtained through a tensile testing for Thermanox™ Coverslips and Elastosil^®^ Film, respectively, and (**C**) the corresponding elastic modulus values calculated as the slope of the linear elastic region (*** *p* < 0.001, *n* = 5). (**D**–**G**) Immunostaining of fibronectin: Thermanox™ Coverslips with FN coating and without, D and E respectively; Elastosil^®^ Films with FN coating and without, F and G respectively (*n* = 2 for each condition).

According to the procedure reported in Materials and Methods Section, the elastic modulus of the two samples was derived from stress-strain curves at a strain percentage of 2% and 20% for Thermanox and Elastosil samples, respectively. In Figure 1A,B, the stress-strain curves of the two samples tested are reported. The elastic modulus measured on Thermanox™ Coverslip is 1350 ± 290 MPa, while for Elastosil^®^ Film is 0.85 ± 0.01 MPa (Figure 1C).

The effectiveness of FN coating was evaluated by immunostaining. Representative images of the staining of FN are reported in Figure 1D–G. No staining was observed in absence of FN (control samples in image C and E), while an homogeneous distribution of protein was detected by polyclonal antibody in both type of FN coated samples (Figure 1D,F).

### 3.2. Effect of Substrates on A549 Cells Morphology and Metabolic Activity

**Figure 2:**
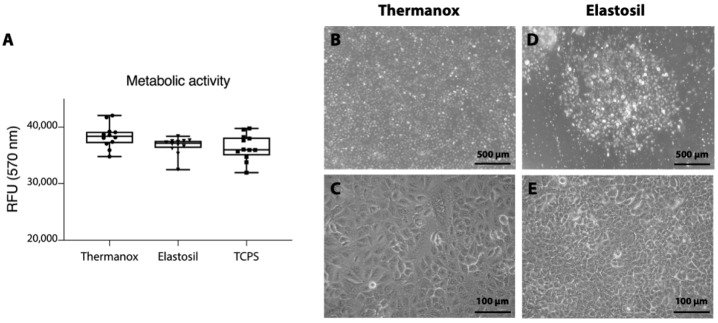
(**A**) Cell metabolic activity (resazurin assay, RFU: relative fluorescence units) of A549 cultured on Thermanox™ Coverslips and on Elastosil^®^ Film (there were no statistical differences between samples, TCPS was used as standard control, *n* = 12). (**B**–**E**) Phase-contrast images of A549 cells cultured on Thermanox™ Coverslips (**B**), magnification 5×; (**C**), magnification 20× or Elastosil^®^ Film (**D**), magnification 5×; (**E**), magnification 20×.

To evaluate the effect of substrate on cell proliferation, A549 cells were cultured on Thermanox™ Coverslips and on Elastosil^®^ Film substrates for 72 h in vitro. The resazurin assay showed that there are no statistically significant differences in the metabolic activity measured on cells seeded on different substrates (Figure 2A). Moreover, in both conditions, the cells displayed a uniform cell adherence. Phase-contrast images (Figure 2B–E) of cultured cells presented differences in cell morphology depending on the substrates. During culture on Thermanox™ Coverslips, the cells spread widely, reaching confluence after 72 h and forming a uniform monolayer covering the available surface (Figure 2B). Otherwise, the cells grown on Elastosil^®^ Film were clearly round and remained distributed in clusters (Figure 2D). The cells did not spread over the membrane surface staying attached in the same position, despite their growth, as it is shown in Supplementary Videos S1 and S2, for Thermanox™ Coverslips and Elastosil^®^ Film respectively.

### 3.3. Scanning Electron Microscopy Analysis

**Figure 3:**
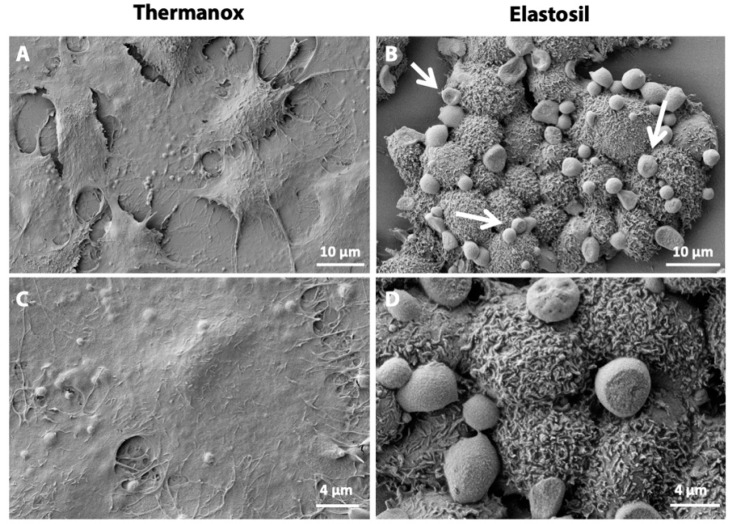
Scanning electron microscopy images of A549 cells cultured on Thermanox™ Coverslips (**A**,**C**) and Elastosil^®^ Film (**B**,**D**) after 72 h of culture. Arrows indicate surfactant on the apical surface of cells.

The morphology and distribution of A549 cells on Thermanox™ Coverslips and Elastosil^®^ Film substrates were examined by SEM analysis (Figure 3). We observed that cells grown on Thermanox™ Coverslips remained flat, expressing a low number of microvilli (Figure 3A,C), while cells cultured on Elastosil^®^ Film maintained the typical alveolar Type II cell morphology, with rounded shape and large number of microvilli on cell surface. In addition, these cells produced an important amount of surfactant on the apical surface (Figure 3B,D), which indicates a healthy monolayer.

### 3.4. Immunofluorescence Analysis for F-Actin, CASP-3 and Focal Adhesion

**Figure 4:**
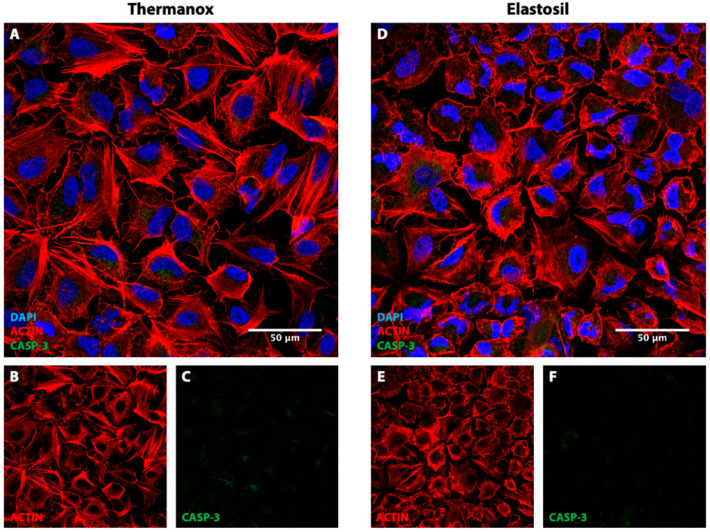
Immunofluorescence images of A549 cells stained for F-Actin (red) and caspase-3 (green) cultured on Thermanox™ Coverslips (**A**–**C**) and on Elastosil^®^ Film (**D**–**F**) after 72 h of culture. Magnification 40× (*n* = 20, representative images are here reported).

F-actin staining revealed that the properties of the substrate importantly affect cells cytoskeleton. The distribution of filamentous actin was visualized by rhodamine-phalloidin staining. As shown in Figure 4A, A549 cells seeded on Thermanox™ Coverslips have a random distribution of F-actin fibers, with a few elongated fibers crossing the entire cellular bodies. In contrast, cells grown up on Elastosil^®^ Film (Figure 4D) displayed a randomly oriented cobblestone shape with actin filaments tightly associated with cell–cell contact, showing a pericellular plasma membrane distribution. These qualitative observations were confirmed by the quantitative investigation performed on immunofluorescence images acquired. The average area of cells cultured on Thermanox substrates was 1892.55 [1186.2–1908.7 IQR] μm^2^ while cells cultured on Elastosil membrane presented an average area of 699.4 [467.7–863.04 IQR] μm^2^.

The staining of caspase-3 was used to evaluate the activity of seeded cells, being CASP-3 a key early indicator of apoptosis. No significant caspase activity was detected in both the seeded substrates, confirming the viable state of the A549 cells. However, a slight increase in the CASP-3 signal was observed in cells seeded on Thermanox™ Coverslips (Figure 4C).

**Figure 5:**
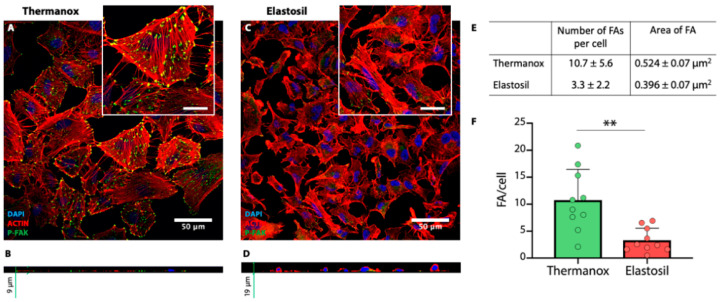
(**A**–**D**) Immunofluorescence images of A549 cells stained for F-actin (red) and focal adhesions (green) cultured on Thermanox™ Coverslips (**A**,**B**) and Elastosil^®^ Film (**C**,**D**) after 72 h of culture (magnification 40×, inset scale bars represent 20 μm). The sections on z-axis are reported in images B and D for Thermanox™ Coverslips and Elastosil^®^ Film respectively. (**E**,**F**) Quantification of focal adhesion observed on Thermanox™ Coverslips and Elastosil^®^ Film (** *p* < 0.01, *n* = 10).

The different distribution of F-actin filaments was also associated with a different expression of focal adhesions identified in A549 cells cultured on Thermanox and Elastosil substrates. FAs are specialized adhesive structures which serve as cellular communication units between cells and the surrounding environment and are directly involved in signal transduction and cytoskeleton organization. As qualitatively reported in Figure 5A–D, the presence of FAs is strongly related to the level of cytoskeleton organization. Cells seeded on Thermanox™ Coverslips exhibited a random distribution of F-actin filaments and a high number of FAs, which resulted less evident on cells seeded on Elastosil^®^ Film. Moreover, the immunofluorescence staining confirmed the cell morphology observed in SEM investigation, where cells seeded on Elastosil membrane assumed a round shape. The acquired sections (z-axis) showed a different profile in cell organization: cells remained round-shaped on Elastosil^®^ Films (19 μm thickness), while appeared flat in a thinner layer on Thermanox™ Coverslips (9 μm thickness) (Figure 5B,D). As a result of FAs quantification, a higher number of FAs per cell was measured on Thermanox™ Coverslips (10.7 ± 5.6) if compared to Elastosil^®^ Films (3.3 ± 2.2). The dimensions of FAs confirmed their mature stage in cells cultured on Thermanox™ Coverslips (Figure 5E,F).

### 3.5. Immunofluorescence Analysis for YAP-1

**Figure 6:**
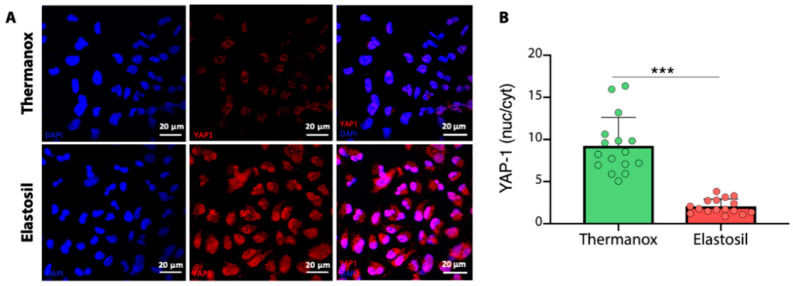
(**A**) Immunofluorescence images of A549 cells stained for YAP-1 (red) cultured on Thermanox™ Coverslips (first row) and on Elastosil^®^ Film (second row). Nuclei are stained in blue. Merged images are shown on the right. Magnification 63× (**B**) Percentage of nuclear YAP-1 in A549 cells cultured on Thermanox™ Coverslips or Elastosil^®^ Film (*** *p* < 0.001, *n* = 16).

To explore the effects of substrate properties on YAP-1 expression, immunofluorescence analysis for YAP-1 was performed. Immunofluorescence showed that a different cytoskeleton organization promoted YAP-1 translocation. YAP-1 was found to be exclusively localized to the nucleus in A549 cells grown on Thermanox™ Coverslips but was predominantly cytoplasmic in cells grown on Elastosil^®^ Film (Figure 6A). Quantification of YAP-1 signal revealed a mean of nuclear/cytoplasmic YAP-1 of 9.24 when A549 cells were cultured on Thermanox™ Coverslips. On the contrary, when cells were cultured on Elastosil^®^ Film the mean of nuclear/cytoplasmic YAP-1 was significantly downregulated to 2.06 (*p* < 0.05) (Figure 6B).

### 3.6. Immunofluorescence Analysis for Surfactant Protein C

**Figure 7:**
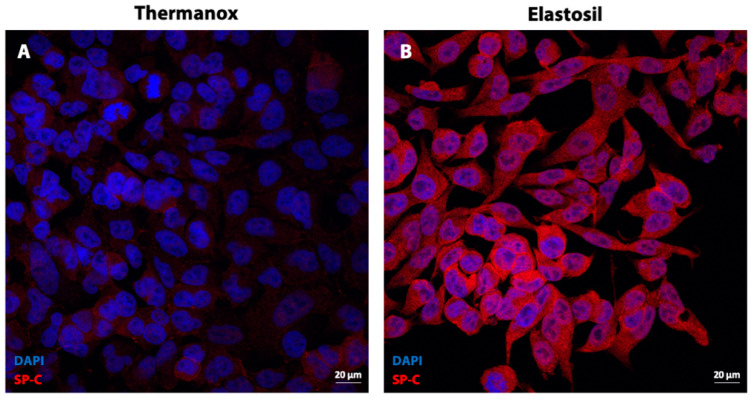
Immunofluorescence images of A549 cells stained for Prosurfactant Protein C (red) and nuclei (blue) cultured on Thermanox™ Coverslips (**A**) and on Elastosil^®^ Film (**B**) after 72 h of culture. Magnification 40×.

In order to investigate the expression of cell type-specific markers, we analyzed surfactant protein C (SP-C) expression. As shown in Figure 7, immunofluorescence staining revealed low-level of cytoplasmic expression of SP-C in A549 cells cultured on Thermanox™ Coverslips. SP-C signal was enhanced on the cytomembrane and in the cytoplasm of A549 cells cultured on Elastosil^®^ Film.

## 4. Discussion

A cell’s surrounding environment is recognized as an important regulator of cell function, as biological cells receive chemical and physical stimuli from their microenvironment. Numerous in vitro studies investigate how physical, chemical and biological aspects of the substrate can influence the functionality of cells seeded on them. In this study, our goal was to assay the effect of two different substrates on the morphology and functions of lung epithelial cells, in order to define more suitable cell culture systems to study specific cell response in vitro.

From the investigation we carried out, substrate characteristics affected cell morphology and cytoskeleton organization of A549 cells, while there were no major differences in the metabolic activity measured on cells seeded on the two substrates considered. Moreover, on both substrates the cells did not show significant caspase activity, confirming the viable state of the A549 cells. In the morphological study, we evidenced that colony formation was different between cells cultured on the two substrates. While the surface layer of fibronectin, acting as an interface between cells and the substrate, is comparable for the two materials, on soft substrate, A549 appear more rounded and less spread than cells seeded on stiffer surfaces. A549 cells on Thermanox™ substrate showed important re-organization of their cytoskeleton with a random but elongated distribution of actin stress fibers spanning almost the entire cell volume, while cells grown on Elastosil^®^ Film substrates exhibited only pericellular distribution of actin stress fibers mostly confined in peripheral plasma membrane. This reorganization of actin fibers may be generated by dynamics of traction forces acting on focal adhesions [36], that are the mechanical link between actin filaments and extracellular matrix. The formation and degradation of actin fibers, and the related focal adhesion sites, are dynamic processes and our results further demonstrate that these processes may be regulated by the density of focal adhesions that in turn may influence cytoskeleton and the related biological pathways with the cell [37,38,39]. Thus, generation of traction forces within the cell cytoskeleton related to the adhesion sites was present more importantly on Thermanox™ Coverslips than on Elastosil^®^ Film. This higher density of focal adhesion site may be responsible for establishing links between actin fibers and the cell substrate, the reorganization of cell cytoskeleton and the related changes in cell biological functions. Despite comparable fibronectin surface density, we cannot exclude that cell culture treated surface of Thermanox™ Coverslips may expose more cell adhesion ligands as compared to Elastosil^®^ Film.

In addition, we also studied how these two different substrates transduced into biological outcomes. The transcriptional coactivators YAP and TAZ are the main downstream mediators of the Hippo pathway. Recent studies have shown the significant role of the Hippo pathway in regulating the differentiation of lung epithelial progenitor cell during embryogenesis. Moreover, YAP and TAZ are considered key regulators of the biological effects due to extracellular matrix rigidity and cell shape [40]. Dupont et al. first reported the relationship between YAP/TAZ activity and ECM stiffness [41]. In cells stretched by a stiff ECM, YAP/TAZ localize predominantly to the nucleus, while they are predominantly cytoplasmic in cells with low levels of mechanical signaling, such as in rounded cells attached to a soft ECM. Their localization is mainly dependent on the tension of the cytoskeleton, however, the mechanisms by which cytoskeletal tension regulates YAP/TAZ remains unclear. Elosegui-Artola et al. have identified a mechanosensing mechanism mediated directly by nuclear pores [42]. Cells exposed to a stiff environment establish a mechanical connection between the nucleus and the cytoskeleton, allowing nuclear flattening [43,44]. This stretches nuclear pores, decreases their sieving function to molecular transport, and increases YAP nuclear import [45]. Our results suggest that a substrate in which focal adhesion sites allow cytoskeleton assembly and organization may lead to nuclear YAP/TAZ localization as we have seen in A549 cells on Thermanox™ substrate, which may induce this mechanosensing mechanism.

Pulmonary surfactant is a mixture of lipids and proteins stored in specialized lysosome-related organelles, called lamellar bodies. It is synthesized exclusively by ATII pneumocytes and secreted into the alveolar space by regulated exocytosis, leading to the reduction of the surface tension at the air/liquid interface preventing the lung collapse. It is generally reported that decreased expression of the surfactant proteins is the main relevant factor in the development of respiratory suffering in prematurely delivered infants [46]. Moreover, people lacking surfactant proteins tend to develop progressive interstitial lung disease [47]. The A549 cells express the surfactant proteins (A, B, C and D). Particularly, SP-C is an ATII integral membrane protein secreted into the alveolar space with surfactant phospholipids where it plays an important role in the formation and maintenance of the surfactant film in alveoli [48]. We observed by SEM analysis an important quantity of surfactant on the apical surface of cells cultured on Elastosil^®^ Film that was not detected in cells grown onto Thermanox™ Coverslips. This was confirmed by immunofluorescence analysis of surfactant-C showing a qualitatively low cytoplasmic expression of SP-C in A549 cells cultured on conventional cell culture substrate while SP-C signal was enhanced in A549 cells cultured on Elastosil^®^ Film. Altogether our results suggest that A549 cells lose the morphology of ATII cells, being flat and without microvilli, and the ability to synthetize surfactant proteins when cultures on a conventional substrate, resembling ATI phenotype. Reduction of surfactant expression in this condition can be due to changes in the state of actin assembly, since actin microfilament system plays an important role in the regulation of surfactant secretion in alveolar epithelial cells, following exocytosis of lamellar bodies [49]. Interestingly, the data on surfactant production together with the morphological changes observed on cells cultured on different substrates strongly suggest that a PDMS membrane (i.e., Elastosil^®^ Film) allows A549 cells maintaining ATII phenotype (i.e., round shape, microvilli, lamellar bodies and surfactant layer), while their culture on standard cell-culture surfaces (i.e., Thermanox™ Coverslips) induce A549 to acquire ATI phenotype. These observations are important for better understanding the yet unknown mechanisms responsible for differentiation of ATII cells in vitro and also in vivo during alveolar tissue disease and repair.

## 5. Conclusions

In summary, we have presented evidence that pulmonary epithelial cell phenotype and functions in vitro are regulated by adhesive properties of the substrate. Our results suggest that two different substrates induced important changes in cell morphology and cytoskeleton organization. Moreover, a standard cell culture substrate led to nuclear YAP/TAZ localization in lung epithelial cells and to a reduction of surfactant C expression, inducing cells to acquire ATI phenotype, while a PDMS substrate induced ATII phenotype. The results from this study bring us a step closer to understanding how cell substrate and the related cell adhesion mechanisms influence morphology and function of alveolar epithelial cells. These results are important for understanding the mechanisms that allow alveolar epithelial cells to retain ATI or ATII phenotype in vitro and also in vivo.

## Data Availability

The data presented in this study are available upon request in Zenodo (https://zenodo.org, accessed on 5 March 2021) at 10.5281/zenodo.4584740.

## Supplementary Materials

The following are available online at https://www.mdpi.com/article/10.3390/biom11050675/s1, Video S1: time lapse of A549 cells seeded on Thermanox^TM^ Coverslip, Video S2: time lapse of A549 cells seeded on Elastosil^®^ Film.
